# Drug delivery system for the extended-release of larotrectinib based on a biocompatible Fe-based metal-organic framework: synthesis, characterization, *in vitro* release properties and antitumor evaluation

**DOI:** 10.3389/fbioe.2023.1197484

**Published:** 2023-06-01

**Authors:** Lu Gan, Peng Ji, Jin-xiang Zhang, Hao Chen, Yan-sheng Yao, Zhen-kun Ren

**Affiliations:** ^1^ The Third Affiliated Hospital of Jinzhou Medical University, Jin Zhou, China; ^2^ Jiangsu Provincial Key Laboratory of Chiral Pharmaceutical Chemicals Biologically Manufacturing, College of Pharmacy and Chemistry & Chemical Engineering, Taizhou University, Taizhou, China; ^3^ The Affiliated Taixing People’s Hospital of Medical College, Yangzhou University, Yangzhou, China

**Keywords:** larotrectinib, metal-organic framework, slow release, biocompatible, antitumor

## Abstract

Larotrectinib (Lar) is an orally administered tropomyosin receptor kinase (Trk) inhibitor with broad-spectrum antitumor activity that is available in clinical dosage forms as capsules and oral solutions. Currently, corresponding research is focused on developing new extended-release formulation systems for Lar. In this study, a biocompatible Fe-based metal-organic framework (Fe-MOF) carrier was synthesized by a solvent-based method, and a sustained-release drug delivery system (Lar@Fe-MOF) was constructed by nanoprecipitation and Lar loading. Lar@Fe-MOF was characterized by transmission electron microscopy (TEM), differential scanning calorimetry (DSC), fourier transform infrared (FTIR) spectroscopy, and thermogravimetric analysis (TGA), and its drug loading capacity and drug release properties were measured by ultraviolet–visible (UV–vis) spectroscopy. Then, the toxicity and biocompatibility of the Fe-MOF carriers were evaluated using 3-(4, 5-dimethylthiazol-2-yl)-2, 5-diphenyltetrazolium bromide (MTT) and hemocompatibility assays. Finally, the anticancer potential of Lar@Fe-MOF was investigated. The TEM results showed that Lar@Fe-MOF had a homogeneous fusiform nanostructural morphology. The DSC and FTIR results showed that Fe-MOF carriers were successfully synthesized and loaded with Lar, which was mainly in an amorphous form. Lar@Fe-MOF showed a large drug loading capacity (–10%) and significant slow-release properties *in vitro*. The MTT assay results showed that Lar@Fe-MOF had good dose-dependent anticancer activity. The *in vivo* pharmacodynamic assay results showed that Fe-MOF significantly increased the anticancer activity of Lar and was biocompatible. In conclusion, the Lar@Fe-MOF system developed in this study is a promising drug delivery platform because it is easy to manufacture, has high biocompatibility and ideal drug release and accumulation, can effectively eliminate tumors with improved safety and is expected to further expand therapeutic applications.

## 1 Introduction

The marketing of larotrectinib (Lar), a broad-spectrum anticancer targeting agent that is effective in treating solid tumors, such as melanoma, colorectal cancer, and breast cancer tumors, for the treatment of adult and pediatric patients with locally advanced or metastatic solid tumors with neurotrophic tyrosine receptor kinase (NTRK) gene fusions was approved by the Food and Drug Administration (FDA) on 26 November 2018 (Berger et al., 2018; Bhangoo and Sigal, 2019).

Currently, Lar is available in two generic formulations, capsules and oral solutions, with limited dosage form options and deficiencies, such as poor stability and low bioavailability. The most significant advantage of extended-release formulations compared with ordinary formulations is the reduced frequency of drug administration. The smooth and slow release of drugs through extended-release technology can prolong the release time, thus constantly maintaining the drug concentration at the effective blood concentration range for a certain period, which can ensure the duration of drug action, reduce the number of doses, and significantly increase patient compliance with the drug (Chaudhary et al., 2019; Ahmed et al., 2020). Therefore, the search for new drug carriers to increase the stability and improve the *in vivo* efficiency of Lar is of great significance for expanding its clinical antitumor applications.

Metal-organic frameworks (MOFs) are novel porous metal-organic hybrid functional materials that have many outstanding advantages over conventional mesoporous materials (Freiberg and Zhu, 2004; Sun et al., 2020), such as 1) high specific surface area and porosity for high therapeutic drug loading; 2) easily modifiable physical (e.g., pore size and shape); and chemical properties through the modification of inorganic clusters and/or organic ligands; 3) open spaces and pores that allow for the interaction of diffusing substrates with binding molecules; 4) moderately strong coordination bonds for biodegradability; and 5) well-defined structures that facilitate the study of host–guest interactions. Due to these unique properties, MOFs are considered one of the best candidates for drug delivery and cancer therapy (Alves et al., 2022; Nikam et al., 2022; Sasmal et al., 2022; Wang et al., 2023).

MOFs have unique properties, such as a highly ordered structure, high specific surface area and large pore capacity, that enable them to adsorb functional molecules onto their outer surface or open channels and trap these molecules within their framework (Shu et al., 2023). MOFs are an ideal class of carrier materials for sustained drug release due to their high drug loading capacity, *in vivo* degradability and ease of modification. These materials undergo several stages of drug release, starting with the dissolution of a portion of the drug on the surface of the material, followed by the progressive diffusion of the drug within the material as the concentration gradient shifts in the direction of the solution and as the drug encapsulated within the material cavity is released by the collapsing framework. In addition, some affinity may exist between a portion of the drug and the material (hydrogen bonding, π-π conjugation, electrostatic adsorption), thus this portion of the drug is released last. As a result of several of these stages occurring continuously, a continuous slow release of the drug is eventually achieved (Freiberg and Zhu, 2004; Leng et al., 2018; Cai et al., 2020). In addition, MOFs can achieve the slow or steady release of loaded drug molecules and have been reported to deliver various chemotherapeutic drugs (Li et al., 2017; Leng et al., 2018; Pettinari et al., 2021). Therefore, MOFs are an excellent carrier material for drug delivery.

In this study, Fe-based metal-organic framework (Fe-MOF) carriers with good biocompatibility, biodegradability, and controlled drug release were synthesized and loaded with Lar to construct a slow drug release system (Lar@Fe-MOF). Lar@Fe-MOF was characterized by transmission electron microscopy (TEM), differential scanning calorimetry (DSC), and other techniques, and its drug loading and release characteristics were evaluated. In addition, murine breast cancer cells and murine erythrocytes were selected to assess the cytotoxicity and biocompatibility of Fe-MOF. Finally, the anticancer potential and *in vivo* toxicity of Lar@Fe-MOF were evaluated at the animal level. Thus, this study provides a new paradigm for expanding Lar extended-release formulations for cancer therapy applications.

## 2 Materials and methods

### 2.1 Materials

Larotrectinib sulfate (Lar), 2-aminoterephthalic acid (BDC-NH_2_), ferrous acetate (FeAc_2_), adipic acid dihydrazide (ADH), N-hydroxysuccinimide (NHS), 1-ethyl-(3-dimethylaminopropyl) carbodiimide hydrochloride (EDC), and N,N-dimethylformamide (DMF) were purchased from Shanghai McLean Biochemical Technology Co., Ltd., China. An MTT assay kit was purchased from China Biyuntian Biotechnology Co., Ltd. Mice (three to four weeks old) were obtained from the Animal Experiment Center of Jinzhou Medical University, China. The animal study protocol was approved by the Animal Ethics Committee of the Jinzhou Medical University of China. All water used for experimentation was double-distilled water.

### 2.2 Synthesis of Fe-MOF

BDC-NH_2_ (0.45 g) was weighed precisely and dissolved in 15 mL of DMF, then aqueous FeAc_2_ solution (0.1 g/mL) was slowly added dropwise to the mixture while stirring, and the reaction was carried out at 65°C for 1–2 h. The solution was cooled naturally at room temperature. The supernatant was separated by centrifugation (6,000 rpm, 30 min) and discarded, and the precipitate was washed twice with DMF (centrifugation, 3,000 rpm, 15 min), followed by two more washes with anhydrous ethanol (centrifugation, 3,000 rpm, 15 min). Finally, the solid precipitate was dried in a vacuum drying oven, and Fe-MOF was obtained (Wan et al., 2019).

### 2.3 Preparation of larotrectinib-loaded nanoparticles

Lar (75 mg) was mixed with 50 mg of Fe-MOF in a 25 mL beaker. The mixture was shaken (200 rpm) for 12 h (Lar@Fe-MOF). The precipitated material was collected after centrifugation (6,000 rpm, 15 min) and dried.

### 2.4 Drug loading of Lar@Fe-MOF

The Lar content was determined by ultraviolet–visible (UV‒vis) spectroscopy. Lar was accurately weighed and added to 3% DMSO and anhydrous ethanol to obtain solutions of different concentrations, which were then filtered through a 0.45 μm nanoporous membrane. The absorbance of different samples was measured at 262 nm to calculate the Lar content and drug loading of Lar@Fe-MOF, and each experiment was repeated three times.

### 2.5 Transmission electron microscopy (TEM) Observations

A small amount of Lar@Fe-MOF was weighed, added to anhydrous ethanol, sonicated, and dispersed for 5 min. A small amount of liquid was dropped onto a copper network, dried, observed by TEM, and imaged (Chen et al., 2020; Chen et al., 2021; Tan et al., 2022).

### 2.6 Differential scanning calorimetry (DSC) analysis

The prepared Lar API, blank Fe-MOF carrier, a Lar and Fe-MOF physical mixture, and Lar@Fe-MOF powder were subjected to DSC. The operating conditions were as follows: an empty aluminum crucible was used as the blank reference, and another crucible was used as the cuvette; 2–5 mg of samples were placed into the cuvette at the corresponding positions for the graphical scans; N_2_ was used as the purge gas, and the heating rate was 10 °C/min. The DSC thermal characteristic curves of each sample were recorded and compared (Gaber et al., 2022).

### 2.7 Fourier transform infrared (FTIR) spectroscopy analysis

The samples (Lar API, blank Fe-MOF carrier, a Lar and Fe-MOF physical mixture, and Lar@Fe-MOF) and an appropriate amount of potassium bromide were mixed in proportion and pressed into tablets. The tablets were then scanned by an FTIR spectroscopy instrument with a wavenumber range of 4,000–400 cm^-1^ and a resolution of 4 cm^-1^ (Lin et al., 2023).

### 2.8 Thermal stability test of Lar@Fe-MOF

Small amounts of Lar API, blank Fe-MOF carrier, and Lar@Fe-MOF samples were warmed up to 400°C under a protective N_2_ atmosphere with a warming rate of 10°C/min to determine their weight loss curves.

### 2.9 Drug release properties

Lar release from Lar@Fe-MOF was studied by performing dialysis in a constant-temperature shaker at 37°C and 100 rpm. Phosphate-buffered saline (PBS, pH 7.4) was chosen as the dialysis medium. Briefly, approximately 5 mg of Lar@Fe-MOF and Lar powder was placed in a dialysis bag, which were then soaked in PBS (100 mL) and tightened at the end. Then, 1 mL aliquots of release medium were removed at different time intervals, with the addition of 1 mL of new release medium to maintain a constant volume. Each group experiment was repeated 3 times. Drug release properties were assessed by evaluating the absorbance of the aliquots using UV‒vis spectroscopy.

### 2.10 Cytotoxicity test of Fe-MOF

Murine breast cancer cells (4T1) were used to assess the biosafety of Fe-MOF. Briefly, 4T1 cells were cultured in Roswell Park Memorial Institute (RPMI) 1640 medium containing 10% fetal bovine serum at 37 °C in a 5% CO_2_ incubator. Cells were inoculated into 96-well plates (density of 1×10^4^) and incubated for 24 h to induce wall attachment. The 96-well plate medium was discarded, and the cells were washed twice with PBS. Media containing different concentrations of Fe-MOF (0.5–50 μg/mL) were then added to each well and incubated for 24 h. Cell viability was determined using the MTT assay according to standard protocols (Javad Farhangi et al., 2021; Liu et al., 2021; Fang et al., 2022; Ji et al., 2022).

### 2.11 Interaction with erythrocytes

The hemocompatibility of the synthesized Fe-MOF was evaluated by analyzing the interaction of Fe-MOF with red blood cells according to a previously reported method (Nikam et al., 2022). Blood was removed from the eyes of mice and placed in tubes containing ethylenediaminetetraacetic acid (EDTA) solution. A certain amount of sodium chloride solution was added to wash the blood cells (centrifuged at 3,500 r/min for 10 min), and then the supernatant was discarded; this procedure was repeated 3 times. The obtained erythrocytes were prepared into a 2% (V/V) suspension with sodium chloride solution and refrigerated at 4°C for further experimentation. The specific experiments were as follows: 0.2 mL of erythrocyte suspension was mixed with 0.8 mL of Fe-MOF suspensions of different concentrations and then incubated at 37°C for 60 min. The supernatant was removed by centrifugation at 3,500 r/min for 10 min. To induce the oxidation of hemoglobin, the collected supernatant was left at room temperature for 10 min. The optical density of oxyhemoglobin was measured at 540 nm, the absorbance of the supernatant was measured, and the percentage of hemolysis was calculated.

### 2.12 *In vitro* antitumor activity evaluation

Briefly, 4T1 cells were added to culture wells and incubated for 24 h to assess the *in vitro* antitumor activity of Lar@Fe-MOF (6.25–100 μg/mL). Follow up as in "2.10".

### 2.13 *In vivo* antitumor activity and toxicity evaluation

The hair around the mammary pads of 3- to 4-week-old female BALB/c mice was shaved off. A primary mammary cancer model was established by implanting 4T1 cells into the hair removal site (Yang et al., 2022). Lar@Fe-MOF preparation was administered orally to environmentally adapted mice at a daily dose of 50 mg/kg for 7 days, and the mice were weighed every other day. General conditions, such as coat color, mental and locomotor abilities, feeding and drinking, as well as signs of intoxication and death, were recorded. By measuring the length of the longest L) and shortest W) axes of the tumor, the tumor volume could be calculated by the formula "V = 1/2 (L × W^2^)". Tumor volumes were monitored every other day. After the last administration, mice in each group were sacrificed on day 8. Mammary tumors and major organs (heart, liver, spleen, lungs, and kidneys) of mice were dissected. The inhibitory effect and potential toxicity were assessed *in vivo* by tumor weighing and hematoxylin and eosin (HE) and terminal deoxynucleotidyl transferase dUTP nick end labeling (TUNEL) staining (Zou et al., 2022; Qu et al., 2023).

### 2.14 Statistical analysis

All statistical tests (mean, standard deviation, and *p*-value) were performed in Excel software, and *p* < 0.05 was considered significant.

## 3 Results and discussion

### 3.1 Construction and characterization of Lar@Fe-MOF

**FIGURE 1:**
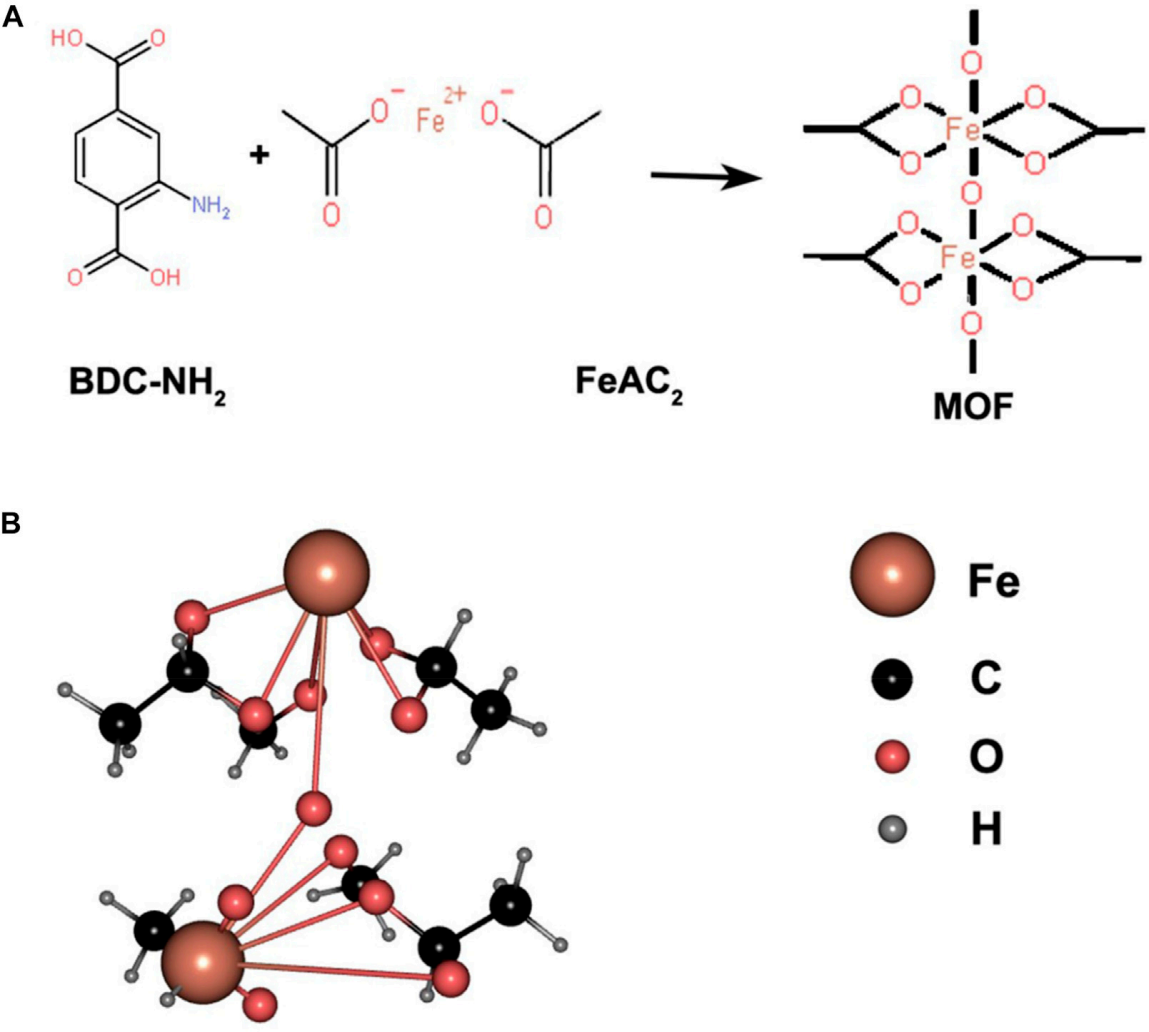
**(A)** Structural formulas of BDC-NH_2_ and FeAc_2_, both ligated to form complexes; **(B)** 3D structure of the formed complex represented by a ball-and-stick model.

MOFs are a class of highly ordered crystalline porous coordination polymers (PCPs). MOFs are considered a promising class of drug nanocarriers due to their obvious structure, high specific surface area and porosity, tunable pore size, and easy chemical functionalization. In recent years, there has been much interest in the study of MOFs for biomedical applications (Sun et al., 2020). A schematic diagram of Fe-MOF synthesis is shown in Figure 1. At high temperatures, Fe^2+^ complexes with BDC-NH_2_ organic ligands form complexes containing multiple coordination bonds (C_8_H_4_NO_4_)nFe, which forms the spatial structure of nanoparticles. Since the Fe^2+^ complexes have multiple -COO- groups, a molecular arrangement with a regular pore structure is formed, conferring porosity to the synthesized organic framework.

#### 3.1.1 Surface morphology and drug loading of Lar@Fe-MOF

**FIGURE 2:**
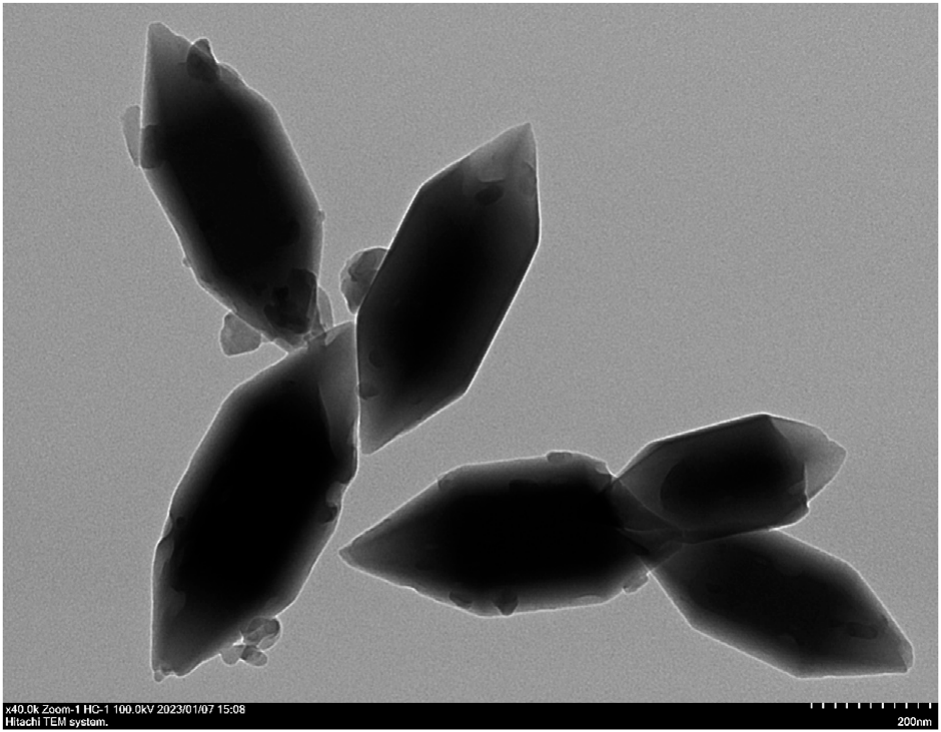
Transmission electron micrographs of Lar@Fe-MOF.

The TEM images showed that Lar@Fe-MOF had a homogeneous fusiform nanostructure morphology and an obvious crystal structure with dimensions of approximately 500 nm in length and 200 nm in width (Figure 2), indicating successful synthesis. The MOF was a porous organic material with good application advantages for drug encapsulation (Guo et al., 2022). The Lar drug loading of Fe-MOF was successfully measured by UV-vis spectroscopy to be 10.3% ± 0.8%. This shows that the Fe-MOF synthesized in this project has significantly improved drug loading capacity, which can effectively enhance drug delivery efficiency and result in sound therapeutic effects (Dhawan et al., 2023).

#### 3.1.2 Differential scanning calorimetry (DSC) results for Lar@Fe-MOF

**FIGURE 3:**
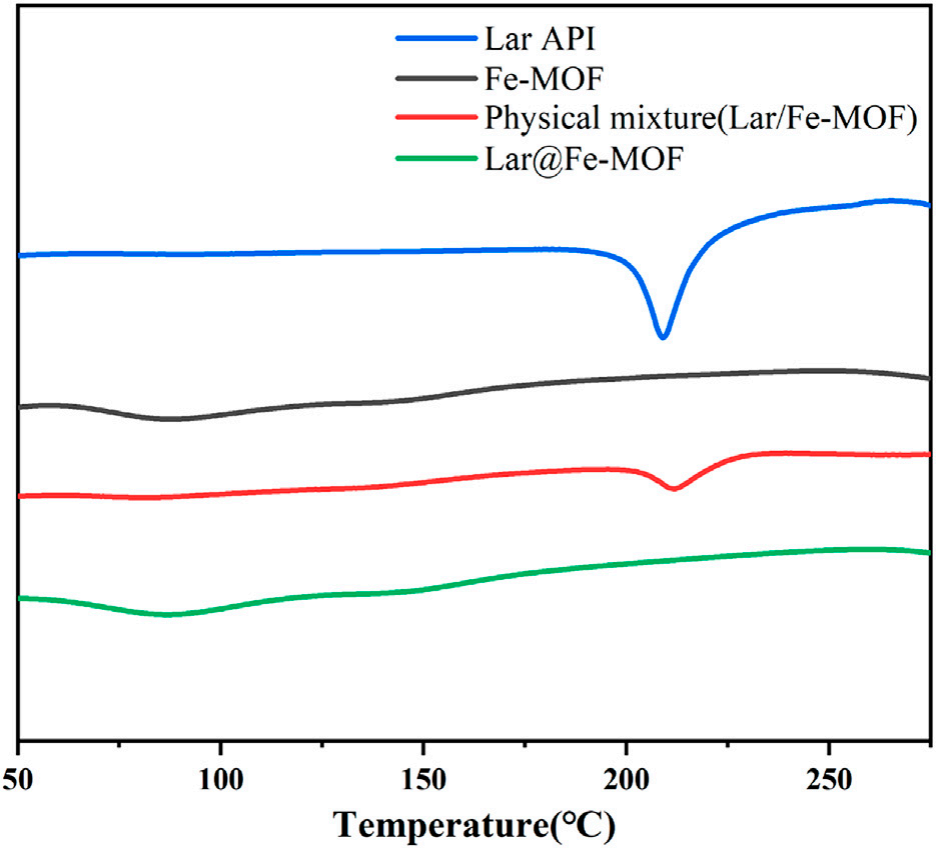
DSC curves of Fe-MOF and Lar@Fe-MOF.

Lar API, blank Fe-MOF carrier, a Lar and Fe-MOF physical mixture, and Lar@Fe-MOF powder were subjected to DSC, and their thermal behaviors are shown in Figure 3. The results showed that Lar exhibited a prominent heat absorption peak at approximately 209°C, indicating that Lar API was crystalline in structure. The physical mixture still had a heat absorption peak at approximately 211°C, indicating that Lar was mixed with the carrier material and that its crystalline form was unaltered. Lar@Fe-MOF did not exhibit a heat absorption peak, and its DSC curve was similar to that of the blank Fe-MOF carrier, probably due to the complete dispersion of the drug in the carrier and its presence in an amorphous form, indicating that Fe-MOF successfully encapsulated Lar (Kujur et al., 2022).

#### 3.1.3 Fourier transform infrared (FTIR) spectroscopy results for Lar@Fe-MOF

**FIGURE 4:**
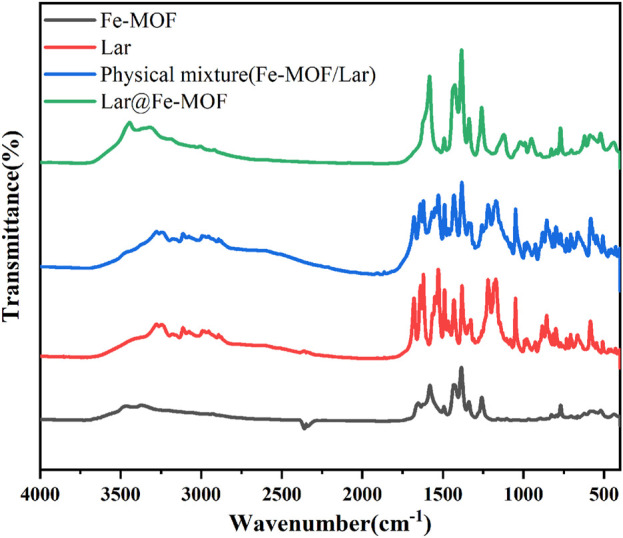
Fourier transforms infrared spectroscopy.

In this experiment, the surface chemical structure of the samples was examined by FTIR spectroscopy. The FTIR spectroscopy results for the blank Fe-MOF carrier, Lar API, the Lar and Fe-MOF physical mixture, and Lar@Fe-MOF are shown in Figure 4. The results shown in the figure indicated that the vibrational peak of the typical carboxyl carbon‒oxygen bond located at ∼1678 cm^-1^ disappeared, which proved that the BDC-NH_2_ organic ligand underwent complete complexation with the Fe ions and successfully formed the Fe-MOF structural framework. The characteristic peaks of Lar API included a C=O stretching vibration peak at ∼1678 cm^-1^ and C-H stretching vibration peaks at ∼2875 cm^-1^ and ∼2992 cm^-1^. The FTIR spectrum of the Lar and Fe-MOF physical mixture was obtained by superimposing the spectra of the two individual components. While the positions of the characteristic absorption peaks of the drug and the material in the drug-loaded nanoparticles (Lar@Fe-MOF) remained the same, the kurtosis became slightly smaller, indicating that the drug was successfully wrapped with or adsorbed into the material (Du et al., 2023; Karimi et al., 2023).

#### 3.1.4 Thermal stability of Lar@Fe-MOF

**FIGURE 5:**
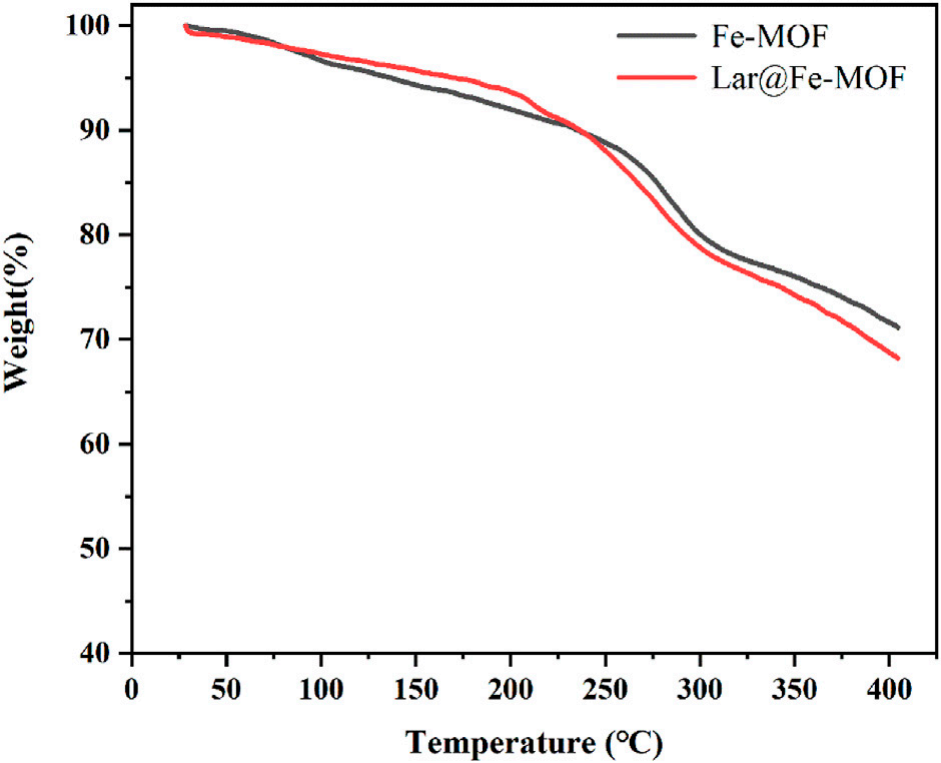
Thermogravimetric curves of Fe-MOF and Lar@Fe-MOF.

The TGA results for Lar@Fe-MOF are shown in Figure 5. As shown in the figure, when the temperature was less than 200°C, the mass loss of Fe-MOF and Lar@Fe-MOF decreased at a low rate of approximately 8%. The difference was not significant, mainly because the mass loss at this stage was primarily the loss of water molecules from the surface and pore channels of the Fe-MOF carrier. No macromolecular degradation occurred (Nikam et al., 2022; Qu et al., 2023). When the temperature increased to 200°C–400°C, the mass loss of Fe-MOF and Lar@Fe-MOF increased, with weight losses of approximately 21.8% and 25.4%, respectively, because of the gradual decomposition of the Fe-MOF matrix. When the temperature was 400°C, the residual masses of Fe-MOF and Lar@Fe-MOF were 71.1% and 68.2%, respectively, mainly because Lar in Lar@Fe-MOF also underwent thermal decomposition when the temperature was higher than 200°C, resulting in a slightly higher final weight loss for Lar@Fe-MOF than for Fe-MOF. In summary, Lar@Fe-MOF exhibited good thermal stability.

### 3.2 *In Vitro* release studies

**FIGURE 6:**
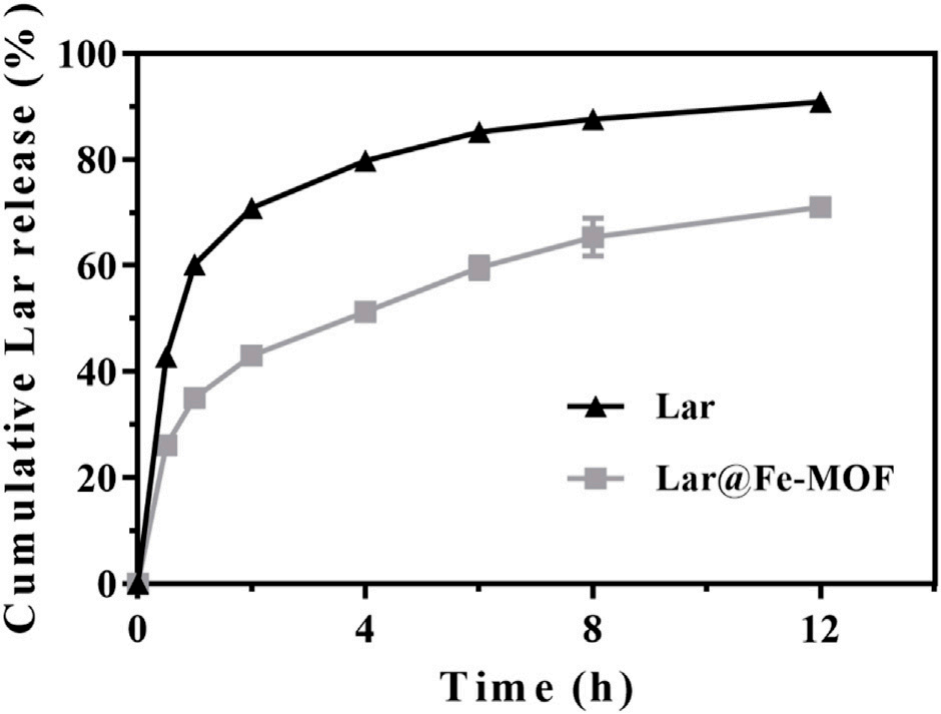
Lar release behavior of Lar@Fe-MOF (*n* = 3).

The *in vitro* release curves (Figure 6) showed that the cumulative release of Lar was time dependent. The main release phase of the Lar solution last for 6 h, with Lar releases of 85.23% at 6 h and 90.91% at 12 h. In contrast, the Lar release of Lar@Fe-MOF reached 59.66% at 6 h, and the cumulative Lar release rate at 12 h was only 71.02%, indicating that Lar@Fe-MOF released Lar more slowly. Thus, the Lar release of Lar@Fe-MOF was much slower, indicating a possible slow-release effect *in vivo*. At present, slow-release controlled release materials are mainly used for drugs that have a short half-life or a low level of oral bioavailability, but which need to be used for a long period of time. The advantage is that the drug can be released at a certain rate over a few hours, weeks or months or even longer to maintain the effective blood concentration and improve bioavailability. Meanwhile, the number of drug administrations is reduced and the toxic side effects of the drug are reduced (Lazar et al., 2023; Shen et al., 2023). Therefore, using Fe-MOF carriers to protect drugs from gastric acid inactivation and to achieve uniform high-concentration drug distribution in various segments of the gastrointestinal tract by prolonged retention and slow release of the anticancer drug Lar through the gastrointestinal tract not only increases patient compliance and improves treatment efficacy, but also reduces the total amount of drug required (Fukumori et al., 2023; Yu et al., 2023).

### 3.3 *In Vitro* cytotoxicity

**FIGURE 7:**
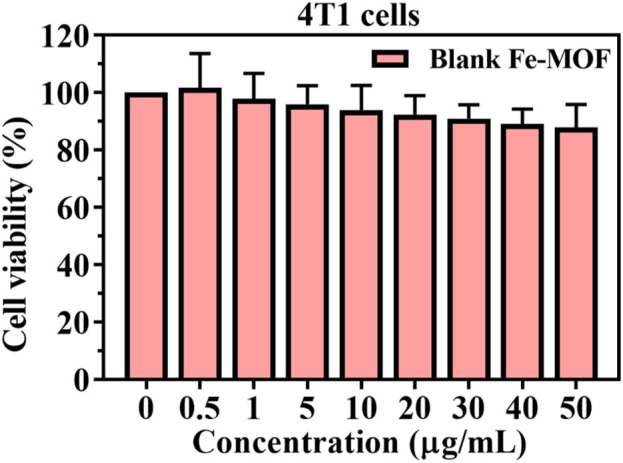
Cell viability of Fe-MOF with 4T1 cells after 24 h incubation (*n* = 3).

To assess the cytotoxicity of the blank Fe-MOF carrier, 4T1 cells were incubated with Fe-MOF (0.5–50 μg/mL) for 24 h. The results are shown in Figure 7. After 24 h, blank Fe-MOF showed no cytotoxic effect and a negligible effect on cell viability. With increasing Fe-MOF concentration, the cell viability gradually decreased, but the cell viability was greater than 85%, indicating that the resulting Fe-MOF carriers had good cytocompatibility (Wang N. et al., 2022; Nikam et al., 2022).

### 3.4 Interaction with erythrocytes

**FIGURE 8:**
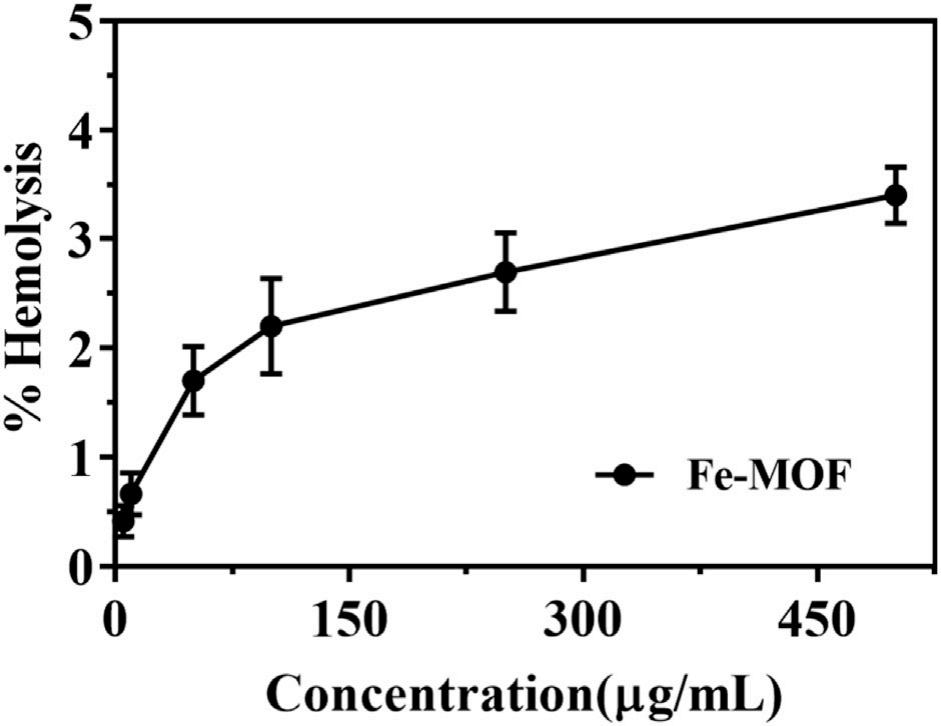
Hemolysis rate of Fe-MOF (*n* = 3).

Studying the interaction between erythrocytes and nanocarriers is essential for the *in vivo* application of nanocarriers (Wang D. et al., 2022; Gong et al., 2022). If the nanocarrier is toxic, it can cause the hemolysis of red blood cells. The results showed that the morphology of erythrocytes was not altered when Fe-MOF, within a mass concentration range of 5–500 μg/mL, was incubated with erythrocytes, and the percentage of hemolysis was less than 5% (Figure 8). Usually, a nanocarrier concentration of no higher than 2 mg/mL is considered a critical safety value (Nikam et al., 2022), which indicated that there was no interaction between the red blood cells and the nanocarrier and that the Fe-MOF carrier had good hemocompatibility and high biosafety.

### 3.5 *In Vitro* antitumor activity evaluation

**FIGURE 9:**
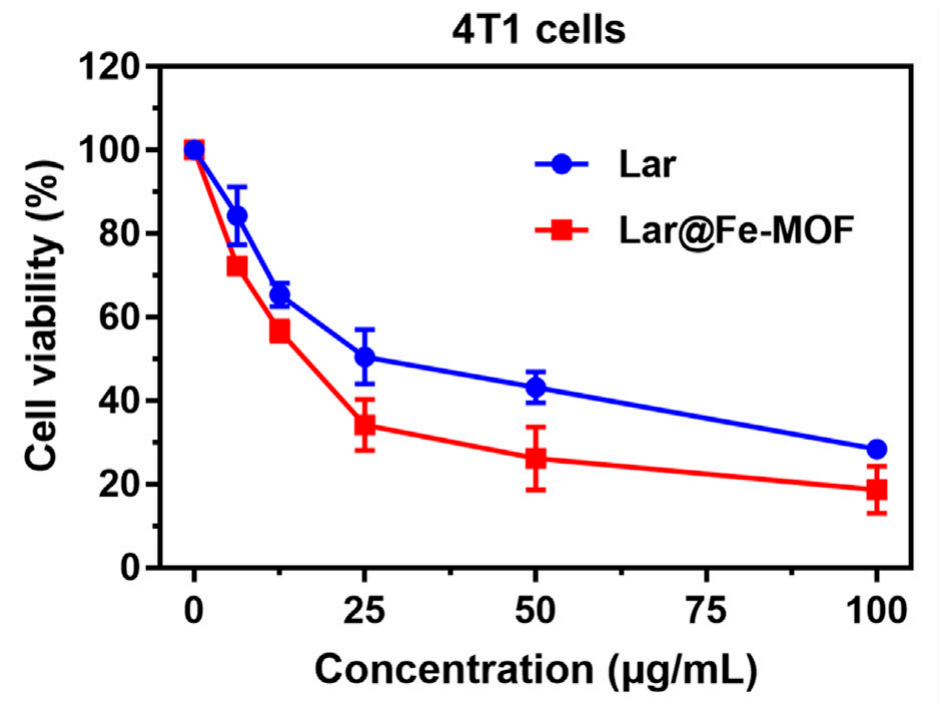
Inhibitory effects of Lar solution and Lar@Fe-MOF solution on 4T1 cells (*n* = 3).

As shown in Figure 9, the cell survival rate of the Lar@Fe-MOF group at 24 h was reduced compared with that of the Lar solution group, indicating that Lar@Fe-MOF enhanced the inhibitory effect of Lar on 4T1 cells. The half-inhibition concentration (IC50) curve was fitted with Graph Pad Prism 7.0. The IC50 values of the Lar solution group and Lar@Fe-MOF group were 14.98 μg/mL and 9.44 μg/mL, respectively. The inhibitory effect of Lar@Fe-MOF on the proliferation of 4T1 cells was stronger than that of the Lar solution group after 24 h of treatment, and the inhibitory effect of Lar@Fe-MOF on the proliferation of 4T1 cells showed a dose-dependent effect, indicating that the cytotoxicity was enhanced by the incorporation of Lar into the Fe-MOF nanoparticles. One reason for this could be the enhanced uptake of the drug by the cells after incorporation into the nanoparticles (Dong et al., 2022; Zhang et al., 2022).

### 3.6 Antitumor effect *in Vivo*

**FIGURE 10:**
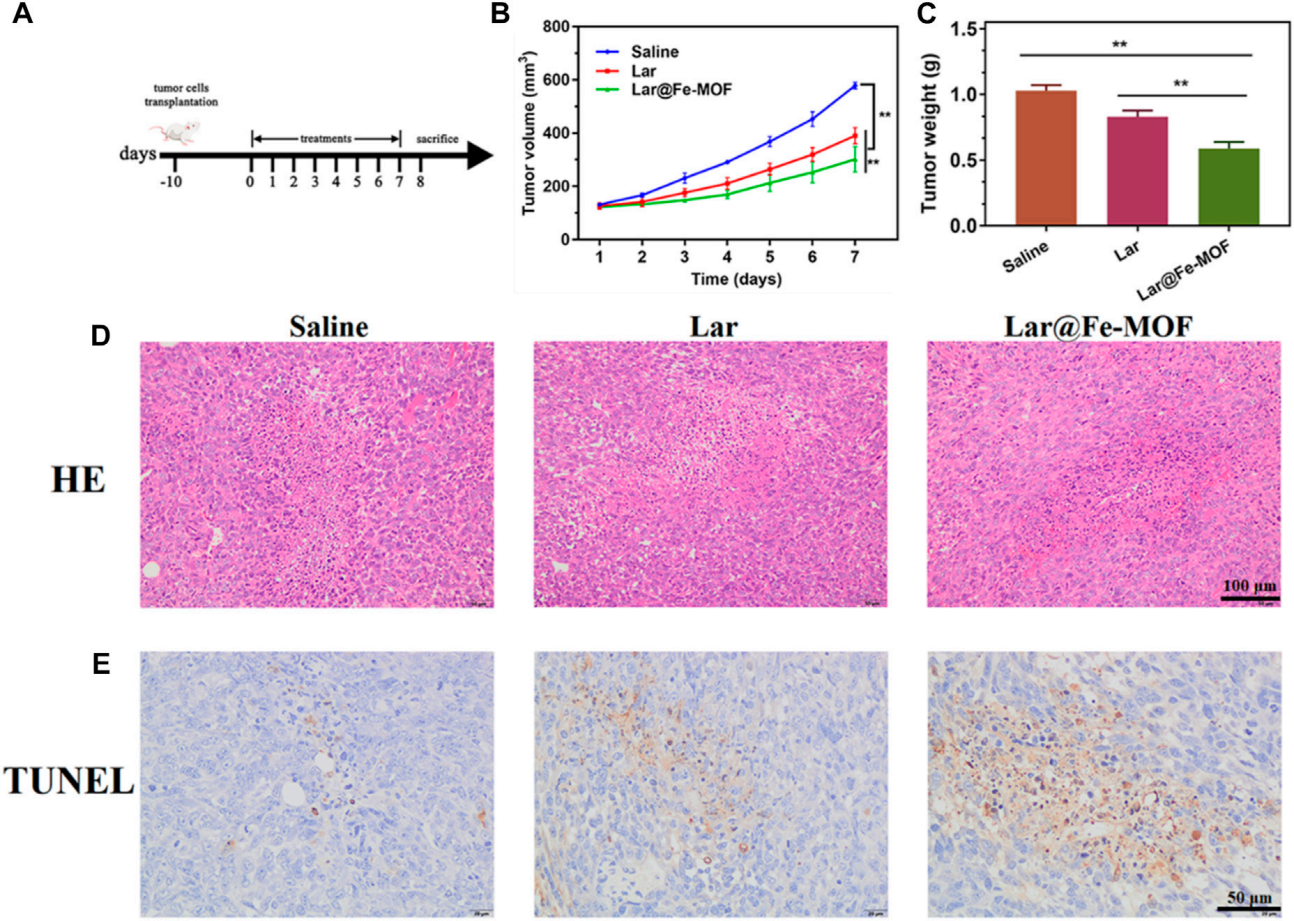
Lar@Fe-MOF exhibited the strongest *in vivo* antitumor efficacy. **(A)** Treatment scheme of saline, Lar, and Lar@Fe-MOF. **(B)** Tumor growth curve with different treatments (n = 5). **(C)** Tumor weights 7 days after the end of treatment (n = 5). **(D)** HE staining of tumors collected 7 days after the end of treatment. **(E)** TUNEL staining of tumors collected 7 days after the end of treatment. Lar@Fe-MOF treatment resulted in the highest apoptotic ratio.

The *in vivo* therapeutic effect of Lar@Fe-MOF was studied in a mouse 4T1 *in situ* breast cancer model. When the tumor volume reached ≈100 mm^3^, the mice were randomly divided into 3 treatment groups (n = 5) and treated with normal saline, Lar solution, and Lar@Fe-MOF for 7 days (Figure 10A). A plot of tumor growth showed that Lar@Fe-MOF resulted in the strongest tumor inhibition (Figure 10B). At day 8, the mean tumor volumes in the Lar and Lar@Fe-MOF groups were 67.5% and 52.1% of those in the saline group, respectively (Figure 10C). Apparently, the tumors were smaller in the Lar@Fe-MOF-treated mice. Cell proliferation in tumors was also significantly inhibited by Lar@Fe-MOF, as shown by the histological results (Figure 10D). TUNEL analysis revealed that Lar@Fe-MOF-treated tumors showed the highest percentage of apoptotic cells (Figure 10E). Thus, Lar@Fe-MOF treatment resulted in a significantly improved *in vivo* antitumor effect in tumor-bearing mice (Chen et al., 2023; Yan et al., 2023).

### 3.7 Safety evaluation

**FIGURE 11:**
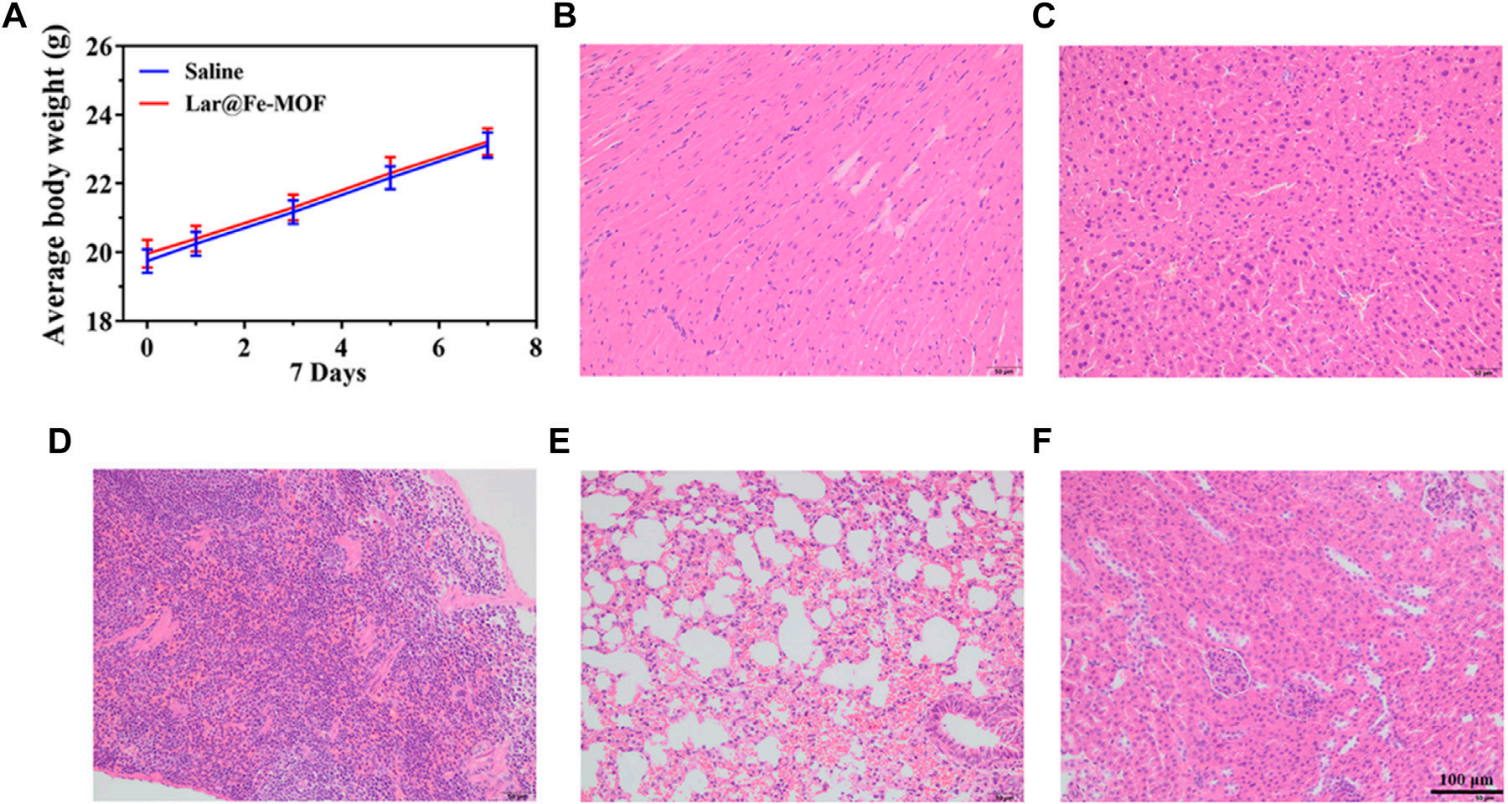
**(A)** Mouse weight recorded every 2 days, and images of HE-stained major organ tissue sections after Lar@Fe-MOF treatment: heart **(B)**, liver **(C)**, spleen **(D)**, lung **(E)**, and kidney **(F)**.

We evaluated the biosafety of Lar@Fe-MOF as this property is a crucial parameter for nanotherapeutic applications in cancer treatment (Ajaz et al., 2022; Jin et al., 2022). After oral administration of Lar@Fe-MOF, the mice showed no abnormal signs of survival and no abnormalities in coat color, diet, water intake, or urinary or fecal conditions. There was no significant difference in the body weight of the mice compared with that in the control group, which showed a normal growth trend (Figure 11A). The mice were dissected after drug administration, and their main organs were visually observed with the naked eye. The internal organs of the mice in each experimental group, including the heart, liver, spleen, lung, and kidney, did not show any apparent lesions. Histopathological sections of the organs, as shown in Figures 11B–F, also indicated that the cells of the tissues did not show noticeable microscopic damage or apparent necrosis and had intact tissue structures and that the organs could remain functionally intact at the administered dose. The above results suggest that Lar@Fe-MOF with excellent biocompatibility can be used as a highly effective oncological treatment strategy with promising applications (Gharehdaghi et al., 2023; Zhao et al., 2023).

## 4 Conclusion

This study investigated new dosage forms based on MOF pharmaceutical carriers. A biocompatible Fe-based metal-organic framework carrier (Fe-MOF) was synthesized by the solvent method. Using larotrectinib (Lar), a broad-spectrum novel anticancer drug, as a model drug, a novel drug delivery system (Lar@Fe-MOF) was prepared. Lar@Fe-MOF was successfully prepared with good stability, as demonstrated by TEM, DSC, and FTIR spectroscopy. *In vitro* release experiments showed that the formulation had prominent slow-release characteristics. *In vitro* toxicity studies showed that Lar@Fe-MOF had good hemocompatibility and low cytotoxicity. *In vivo* toxicity studies showed that mice did not show significant liver and kidney toxicity at the administered dose. *In vitro* and *in vivo* pharmacodynamic studies demonstrated the enhanced antitumor activity of Lar@Fe-MOF. These results suggest that Fe-MOF is a promising new biocompatible carrier that provides a new approach for the sustained-release of Lar and can be used as a safe biomaterial for *in vivo* drug delivery and further studies.

## Data Availability

The original contributions presented in the study are included in the article/supplementary material, further inquiries can be directed to the corresponding authors.
